# West Ham Hospital

**Published:** 1902-06-28

**Authors:** 


### WEST HAM HOSPITAL.

### THE YEAR'S WORK.

There are now four wards in this hospital, containing 60 beds; 28 are for male patients, 13 for female, and there are 19 cots for children. There are three medical officers, besides a surgeon-dentist, an ophthalmic surgeon, and a senior and junior house surgeon. The nursing staff is composed of the matron, four sisters, three staff nurses, and 14 probationers. Of 961 in-patients treated during 1901, 771 were surgical and 190 medical; 393 were discharged cured, 448 relieved, 75 died, and 45 remained in the hospital at the close of [illegible]e year. Most of the patients came from "London over the border," covering the large district between the London Hospital on one side and Poplar Hospital on the other. The average stay was 18 days, and 46 was the average daily number of patients. The figures in the previous year were 22 and 42 respectively. There was an increase of over 35 per cent. of in-patients, and of nearly 15 per cent. of out-patients' attendances, as compared with 1900. The cost of each in-patient was £1 12s. 4d. per week, a decrease of nearly 9 per cent. Subscriptions were £930, donations £872. A very gratifying legacy was received from the trustees of Mr. G. J. Lawrence, whose property was left to the best-managed charities in the southern half of the county of Essex; one-sixth = £2,738 was awarded to this hospital after careful investigation. King Edward's Fund gave an annual grant of £300, and a special grant of £600, the Sunday Fund gave £375, and the Saturday Fund £124 and a box collection of 17s. 9d. Workpeople's contributions were £366. The total ordinary income was £5,271; extraordinary, £2,769. The total ordinary expenditure was £5,475; extraordinary, £574. The sum of £2,691 was invested in West Ham Corporation stock and Consols. The Samaritan Fund is sadly in need of help.

